# α-Tocopherol Modulates Non-Amyloidogenic Pathway and Autophagy in an In Vitro Model of Alzheimer’s Disease: A Transcriptional Study

**DOI:** 10.3390/brainsci9080196

**Published:** 2019-08-10

**Authors:** Agnese Gugliandolo, Luigi Chiricosta, Serena Silvestro, Placido Bramanti, Emanuela Mazzon

**Affiliations:** IRCCS Centro Neurolesi “Bonino-Pulejo”, Via Provinciale Palermo, Contrada Casazza, 98124 Messina, Italy

**Keywords:** in vitro model, α-tocopherol, Alzheimer’s disease, amyloid β, autophagy

## Abstract

Alzheimer’s disease (AD) is the most common form of dementia worldwide. The hallmarks of AD are the extracellular amyloid plaques, which are formed by amyloid β (Aβ) aggregates derived from the processing of the amyloid precursor protein (APP), and the intraneuronal neurofibrillary tangles, which are formed by the hyperphosphorylated tau protein. The aim of this work was to study the effects of α-tocopherol in retinoic acid differentiated SH-SY5Y neuroblastoma cells exposed to Aβ_1-42_ evaluating the transcriptional profile by next-generation sequencing. We observed that α-tocopherol was able to reduce the cytotoxicity induced by Aβ treatment, as demonstrated by Thiazolyl Blue Tetrazolium Bromide (MTT) assay. Moreover, the transcriptomic analysis evidenced that α-tocopherol treatment upregulated genes involved in the non-amyloidogenic processing of APP, while it downregulated the amyloidogenic pathway. Moreover, α-tocopherol modulated the expression of the genes involved in autophagy and the cell cycle, which are both known to be altered in AD. The treatment with α-tocopherol was also able to reduce oxidative stress, restoring nuclear factor erythroid-derived 2-like 2 (Nrf2) and decreasing inducible nitric oxide synthase (iNOS) levels, as demonstrated by immunocytochemistry.

## 1. Introduction

Alzheimer’s disease (AD) is a progressive neurodegenerative disorder characterized by cognitive and functional deficits, and in particular defects in short-term memory [1]. It represents the most common type of dementia. Electroencephalography (EEG) and different tests, such as the Mini Mental State Examination allow the detection of patients affected by AD and the differentiation between different types of dementia [2,3,4]. The number of patients affected by AD is increasing and until now, no efficacious cure that was able to slow disease progression has been available. For these reasons, AD represents one of the most problematic and costly illnesses.

The hallmarks of AD from a molecular point of view are extracellular amyloid plaques and intraneuronal neurofibrillary tangles (NFTs) [1]. Their accumulation leads to the degeneration and death of neurons in AD.

Amyloid plaques are composed of amyloid β (Aβ) aggregates that derived from the cleavage of the amyloid precursor protein (APP). APP can be processed through the amyloidogenic or the non-amyloidogenic pathway. Specifically, APP is mainly processed through the non-amyloidogenic pathway, where the α-secretase cleaved APP producing soluble APPα (sAPPα) and the 83-amino acid C-terminal fragment (C83). In turn, it can be cleaved by the γ-secretase to produce two small fragments, p3 and the APP intracellular domain (AICD). When APP follows the amyloidogenic pathway, APP is cleaved by the β-secretase β-site APP-cleaving enzyme 1 (BACE1), generating the soluble APPβ (sAPPβ) metabolite and the β-carboxyl terminal fragment (CTFβ or C99). CTFβ is in turn cleaved by γ-secretase to finally produce Aβ peptides and the AICD fragment. Interestingly, APP cleavage by α-secretase inhibits the formation of Aβ, given that the cleavage site is inside the Aβ domain [5]. Among the various Aβ isoforms, Aβ_1-40_ and Aβ_1-42_ are the most important ones [6]. However, Aβ_1-42_ is more prone to aggregation compared to the other Aβ peptides, and for this reason, it is mainly linked to the neurodegeneration observed in AD [6,7].

AD is also characterized by the presence of the hyperphosphorylated tau protein, which is a key microtubule-associated protein. Insoluble aggregates of tau protein can impair axonal transport, causing mitochondria dysfunction and neuronal damage [8].

Different pathological processes are involved in AD, such as oxidative stress, neuroinflammation, and mitochondrial and autophagy dysfunctions [9,10,11]. Autophagy is a catabolic process that involves the sequestration of protein aggregates or injured cell organelles into the autophagosome, which is formed by a double membrane, and its degradation after the fusion with a lysosome. After the degradation, the formed amino acids, nucleotides, and the other components can be recycled [12]. However, the autophagosomes can represent a reservoir of Aβ, and for this reason, dysfunctions of autophagy may enhance Aβ accumulation and toxicity [13].

Evidence indicated lower plasma and cerebrospinal fluid levels of vitamin E in AD patients [14,15], while higher plasma levels seem to lower AD risk [16]. The term vitamin E is used to indicate eight different lipid-soluble compounds differentiated on the bases of the number and position of the methyl groups on the chromanol rings, which are termed α-, β-, γ-, and δ-tocopherols and tocotrienols. The α-tocopherol is the form that shows the highest concentration in human tissues [17]. It is a well-known antioxidant, but it is also able to influence enzyme activity, as well as modulate signal transduction and gene regulation [17,18,19]. Vitamin E is really important for neurons; indeed, its deficiency caused cellular atrophy and reduced the dendritic branching of Purkinje neurons, which is associated with motor coordination and cognitive deficits. However, vitamin E supplementation normalized behavioral and cognitive functions [20]. Interestingly, vitamin E was shown to be able to counteract oxidative stress and toxicity induced by Aβ and to exert beneficial effects in AD animal models [21].

However, even if vitamin E exerts antioxidant and neuroprotective actions, its effectiveness in treating AD is not clear. Evidence showed a partial efficacy of vitamin E in slowing the progression of dementia. Indeed, some studies showed that vitamin E treatment gave positive results on cognition, but in others, it was not able to delay AD. However, the differences in the nutritional status of the patients, the antioxidant effect of vitamin E in each person, and the range of time of brain compensation in each person may be some of the reasons that explain the different results regarding vitamin E efficacy [22].

The aim of this study was to evaluate the transcriptional changes induced by α-tocopherol treatment in retinoic acid (RA)-differentiated neuroblastoma SH-SY5Y cells exposed to Aβ_1-42_.

## 2. Materials and Methods

### 2.1. Cell Culture and Differentiation

The human neuroblastoma cell line SH-SY5Y was obtained from American Type Culture Collection (ATCC) (Manassas, VA, USA). The cells were grown in a monolayer at 37 °C in 5% CO_2_ humidified atmosphere in Dulbecco’s Modified Eagle’s Medium/Nutrient Mixture F-12 Ham (DMEM/F12) medium (Sigma-Aldrich, Saint Louis, MO, USA) supplemented with 10% fetal bovine serum (FBS) (Sigma-Aldrich). In order to induce differentiation, SH-SY5Y cells were exposed to 10 µM of RA (Sigma-Aldrich) for 5 days.

### 2.2. Cell Treatment with Aβ_1-42_ and α-Tocopherol

Aβ_1-42_ (Sigma-Aldrich, Saint Louis, MO, USA) was dissolved in dimethyl sulfoxide (DMSO), diluted in phosphate buffered saline (PBS), aggregated at 37 °C for 24 h, and added to the medium at the final concentration (final DMSO concentration was <0.1%). A previous work demonstrated that Aβ_1-42_ incubated for 24 h at 37 °C formed aggregates [23]. Cells were pre-treated with α-tocopherol for 24 h. The day after, cells were treated with the medium containing 10 µM of Aβ_1-42_, α-tocopherol alone, or 10 µM of Aβ_1-42_ with α-tocopherol for 24 h. The concentration of Aβ_1-42_ was chosen on the basis of previous studies available in the literature indicating that it was able to exert cytotoxicity in SH-SY5Y cells [24,25,26,27]. Control cells were incubated with DMEM/F12 medium supplemented with 10% FBS. In order to evaluate cell viability, different α-tocopherol concentrations were tested (10 µM, 50 µM, 100 µM, and 200 µM).

### 2.3. Cell Viability

Cell viability in the presence of different concentrations of α-tocopherol and Aβ_1-42_ was evaluated using the Thiazolyl Blue Tetrazolium Bromide (MTT) assay. SH-SY5Y cells were cultured in 96-well plates, differentiated for 5 days with RA, and treated with α-tocopherol and Aβ_1-42_, as reported in the previous paragraph. At the end of the treatment, cells were incubated with medium containing MTT (0.5 mg/mL; Sigma-Aldrich) at 37 °C for 4 h. The formed formazan crystals were dissolved in acidic isopropanol at 37 °C for 1 h, and the optical density was evaluated by the spectrophotometric measurement of absorbance using the microplate reader Victor NIVO^TM^ (PerkinElmer, Waltham, MA, USA). The assay was repeated in triplicate.

### 2.4. Extraction of Total RNA and cDNA Library Preparation

RNA was extracted using the Maxwell® RSC simplyRNA Cells Kit (Promega, Madison, Wisconsin, USA) following the manufacturer’s instruction. The library preparation was carried out according to the TruSeq RNA Exome protocol (Illumina, San Diego, CA, USA) as previously described [28]. The experiment was repeated in triplicate.

### 2.5. RNA-Seq Data Analysis and Gene Evaluation

In order to perform the cDNA analysis, differentiated SH-SY5Y treated with Aβ (SH-SY5Y-Aβ) and cells treated with α-tocopherol and Aβ (SH-SY5Y-Aβ-αT) were sequenced by a MiSeq Next-Generation Sequencing instrument (Illumina, San Diego, California, USA). The MiSeq output files in “bcl” format were demultiplexed by CASAVA software (version 1.8) (Illumina) in “Fastq” format files. The software fastQC (Babraham Institute, Cambridge, UK) was used to perform the quality check of the files. Adapters and low-quality bases were removed by Trimmomatic (Usadel Lab, Aachen, Germany) [29] (version 0.38) (LEADING: 30 TRAILING: 28 SLIDINGWINDOW: 4:28 MINLEN: 75). The alignments of the reads were made against the “Homo Sapiens” reference genome available on the University of California Santa Cruz (UCSC) website [30] by Spliced Transcripts Alignment to a Reference (STAR) RNA-seq aligner [31]. Then, the alignment was sorted by the same program. The changes in the expression profile between SH-SY5Y-Aβ versus SH-SY5Y-Aβ-αT were studied with Cuffdiff software (Trapnell lab, Washington, USA) version 2.2.1 [32], taking advantage of the Homo Sapiens GTF file still available on the UCSC website. The Kyoto Encyclopedia of Genes and Genomes (KEGG) database [33,34] was used to inspect the role of the genes involved in the Alzheimer’s disease pathway (hsa05010), in the oxidative phosphorylation pathway (hsa00190), in the cell cycle pathway (hsa04110), and in the autophagy–animal pathway (hsa04140).

### 2.6. Immunocytochemistry

SH-SY5Y cells were grown on coverslips (12 mm; Thermo Fisher Scientific, Waltham, MA, USA) and treated with Aβ_1-42_ and α-tocopherol, as reported in the previous paragraph. At the end of the treatment, cells were fixed with 4% paraformaldehyde at room temperature for 30 min. Afterwards, cells were washed with PBS (pH 7.5) and incubated with 3% H_2_O_2_ at room temperature for 15 min, with the aim to suppress the endogenous peroxidase activity. SH-SY5Y cells were washed three times with PBS, and they were blocked with horse serum +0.1% Triton X-100 for 20 min. Afterwards, cells were incubated overnight at 4 °C with the primary antibodies anti-inducible nitric oxide synthase (iNOS) (1:50; Santa Cruz Biotechnology) and anti-nuclear factor erythroid-derived 2-like 2 (Nrf2) (1:50; Santa Cruz Biotechnology). Afterwards, cells were washed with PBS and incubated with biotinylated secondary antibody (1:200; Vector Laboratories, Inc., Burlingame, CA, USA) and streptavidin AB Complex-horseradish peroxidase (HRP) (ABC-kit from Dako, Glostrup, Denmark). The immunostaining was developed with the 3,3′-Diaminobenzidine (DAB) peroxidase substrate kit (Vector Laboratories, DBA Italia S.r.l., Milan, Italy; brown color; positive staining) and counterstaining with nuclear fast red (Vector Laboratories, DBA Italia S.r.l., Milan, Italy; pink background; negative staining). The immunocytochemical assays were repeated three times, and each experimental group was plated in duplicate, for a total of six coverslips for each antibody. The images were captured using a light microscopy (LEICA DM 2000 combined with LEICA ICC50 HD camera; Leica, Wetzlar, Germania).

### 2.7. Statistical Analysis

Statistical analysis of cell viability was carried out using GraphPad Prism version 7.0 software (GraphPad Software, La Jolla, CA, USA). The multiple comparison was performed using a one-way ANOVA test and the Bonferroni post hoc test. A *p* value less than or equal to 0.05 was considered statistically significant. The results are expressed by mean ± standard deviation (SD).

## 3. Results

### 3.1. Cell Viability

**Figure 1:**
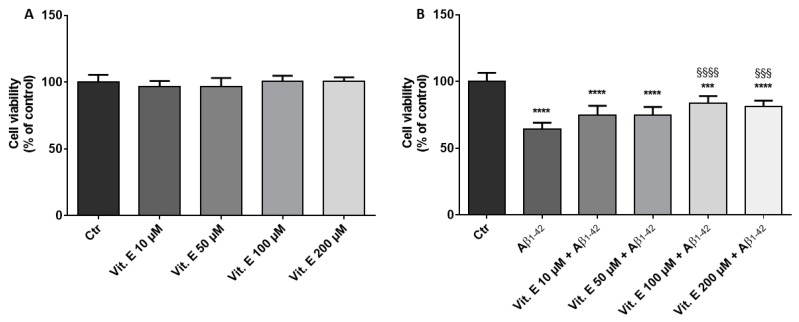
Evaluation of cell viability in differentiated SH-SY5Y treated with α-tocopherol and/or Aβ_1-42_ using MTT assay. (**A**) α-tocopherol treatment did not cause a reduction in cell viability. (**B**) 100 µM of α-tocopherol was able to counteract the loss of cell viability induced by Aβ_1-42_ treatment. *** *p* < 0.001, **** *p* < 0.0001 compared to the control cells; ^§§§^ *p* < 0.001, ^§§§§^ *p* < 0.0001 compared to Aβ_1-42_ treated cells.

The results of the MTT assay evidenced that α-tocopherol treatment did not cause cytotoxicity in the range 10 to 200 µM (Figure 1A). On the contrary, the treatment of RA-differentiated SH-SY5Y with 10 µM of Aβ_1-42_ for 24 h caused a reduction of cell viability. However, 100 µM of α-tocopherol was able to significantly increase cell viability compared to Aβ_1-42_ treated cells (Figure 1B). Lower doses of α-tocopherol, although increasing the percentage of cell viability, did not induce a significant increase. The highest dose of α-tocopherol tested did not increase the protective effect against Aβ_1-42_. On the basis of these results, we performed the transcriptomic analysis using the α-tocopherol concentration of 100 µM.

### 3.2. Gene Inspection

The RNA-Seq analysis was performed by Cuffdiff software. The comparison between SH-SY5Y-Aβ versus SH-SY5Y-Aβ-αT samples revealed 11,907 genes, of which 1966 genes were statistically different in their expression levels (q < 0.05). Regarding the significant genes, 971 genes were upregulated in SH-SY5Y-Aβ-αT, while 995 were downregulated. The Alzheimer’s disease pathway revealed that 11 genes were upregulated and five genes were downregulated in SH-SY5Y-Aβ-αT compared to SH-SY5Y-Aβ (Table 1).

The same comparison highlighted that in the oxidative phosphorylation pathway, 10 genes were upregulated, while four genes were downregulated in SH-SY5Y-Aβ-αT compared to SH-SY5Y-Aβ (Table 2).

The cell cycle pathway showed that eight genes were upregulated and 16 genes were downregulated in SH-SY5Y-Aβ-αT compared to SH-SY5Y-Aβ (Table 3).

**Figure 2:**
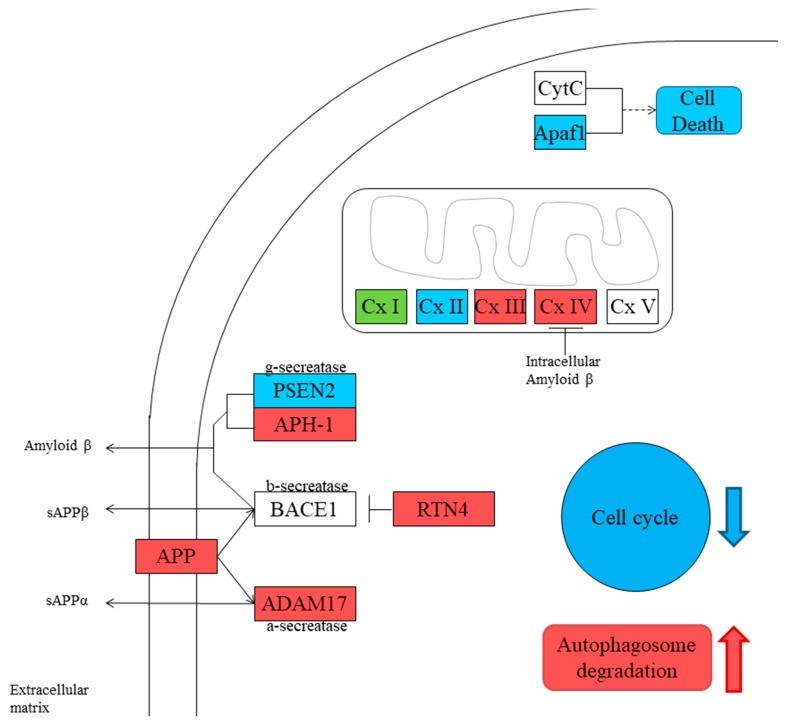
Distribution of the genes significantly modulated in the Alzheimer’s disease pathway. The red background represents proteins encoded by upregulated genes in SH-SY5Y-Aβ-αT. The blue background represents proteins encoded by downregulated genes in SH-SY5Y-Aβ-αT. The green background represents proteins encoded by both upregulated and downregulated genes in SH-SY5Y-Aβ-αT. The white background indicates that there was no statistical difference in the level of gene expression. *APP*, amyloid precursor protein; *ADAM17*, ADAM metallopeptidase domain 17; *RTN4*, Reticulon 4; *PSEN2*, presenilin 2; *APH1B*, aph-1 homolog B, gamma-secretase subunit; *APAF1*, apoptotic peptidase activating factor 1. *BACE*, beta-secretase 1; CytC, cytochrome C; Cx I, complex I; Cx II, complex II; Cx III, complex III; Cx IV, complex IV; Cx V, complex V.

**Figure 3:**
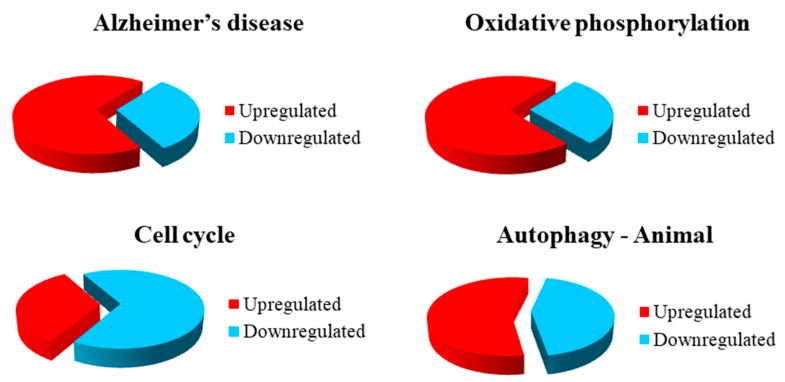
Distribution of gene expression among the different KEGG pathways.

In the autophagy–animal pathway, 16 genes took part, of which nine genes were upregulated and seven genes were downregulated in SH-SY5Y-Aβ-αT (Table 4). The representation of the genes in the pathways is shown in Figure 2, while the summing of the changes in gene expression is represented in Figure 3.

### 3.3. Immunocytochemistry for iNOS and Nrf2

**Figure 4:**
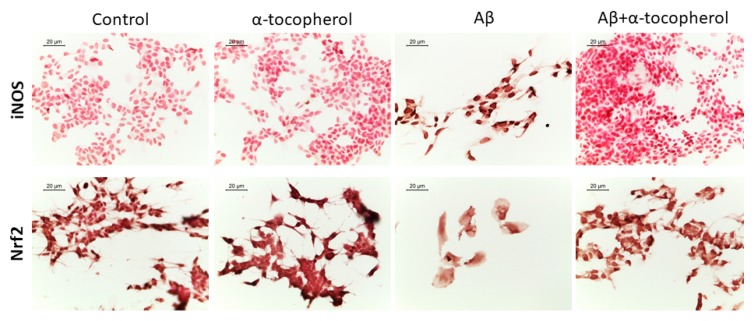
Immunocytochemistry for inducible nitric oxide synthase (iNOS) and anti-nuclear factor erythroid-derived 2-like 2 (Nrf2). The evaluation of iNOS and Nrf2 evidenced the presence of oxidative stress in Aβ-treated SH-SY5Y cells, while α-tocopherol was able to restore their protein levels. Objective: 40×, scale bar: 20 µm.

Given the antioxidant capacity of α-tocopherol, we also evaluated markers of oxidative stress by immunocytochemistry. We observed an increase of iNOS and a reduction of Nrf2 in Aβ-treated SH-SY5Y cells. However, the treatment with α-tocopherol was able to restore Nrf2 levels and decrease iNOS protein levels (Figure 4).

## 4. Discussion

Previous reports in vitro and in vivo indicated the ability of vitamin E to reduce the toxicity of Aβ peptides. In particular, vitamin E was able to reduce oxidative stress and cognitive deficits [21]. Given the promising results obtained in experimental models, clinical trials evaluated vitamin E supplementation in AD patients, but only some of them indicated improvements, showing that vitamin E was able to slow AD progression and cognitive decline [21,35,36].

In this study, we aimed to characterize the changes in the transcriptional profile induced by α-tocopherol in SH-SY5Y cells treated with Aβ using next generation sequencing (NGS) technology and focusing on pathways known to be altered in AD.

We observed that the treatment with α-tocopherol was able to increase the processing of APP through the non-amyloidogenic pathway. We observed the increased expression of *APP* and ADAM metallopeptidase domain 17 (*ADAM17)* genes. *ADAM17* encodes for α-secretase, that as said in the introduction, processes APP leading to the production of sAPPα. This result is in line with a previous work reporting that carnosic acid—a compound derived from rosemary that is able to exert an antioxidant action activating Nrf2—increased the mRNA expression of ADAM17, but only marginally increased the mRNA expression of ADAM metallopeptidase domain 10 (ADAM10), suggesting that it reduces Aβ production by activating ADAM17 in human neuroblastoma cells [37]. On the contrary, the processing of APP through the amyloidogenic pathway seemed to be reduced. Indeed, we found the upregulation of Reticulon 4 (*RTN4)*, while the expression of presenilin 2 (*PSEN2)*, part of the γ-secretase, was reduced. RTN4 is known to inhibit the β-secretase action reducing the production of Aβ [38,39]. However, aph-1 homolog B, gamma-secretase subunit (*APH1B)* was increased, but it also has a role in inhibiting apoptosis [40,41]. These results may indicate the increased production of sAPPα, which showed neuroprotective and pro-survival effects, and has a role in promoting neurogenesis [42,43,44]. Different stress conditions induce APP upregulation, given its neuroprotective and neurotrophic roles. Indeed, APP maintains synapses and plays a role in neuronal survival [9]. The induction of α-secretase activity exerts a neuroprotective effect against AD, thanks to its ability to promote some neurotrophic signaling pathways; for this reason, it may be a new strategy to reduce Aβ [45].

AD is characterized by mitochondrial dysfunctions [46], and mitochondria represent a site where Aβ is accumulated in AD neurons [47]. It was reported that Aβ altered the function of some proteins of mitochondrial oxidative phosphorylation [48]. We found that α-tocopherol treatment was able to increase the expression of the genes encoding for proteins that take part in Complex I (*NDUFB1, NDUFB3, NDUFC2, NDUFS4*), Complex III (*UQCRB, UQCRH*), and Complex IV (*COX7A2, COX10*). Only the genes *NDUFA1* and *NDUFA6*, which encoded for subunits of Complex I, and *SDHA*, encoding for Complex II, were downregulated. These results may indicate a better functioning of mitochondrial complexes, and that α-tocopherol may counteract the toxicity induced by Aβ in mitochondrial oxidative phosphorylation. Aβ-induced mitochondrial dysfunctions are linked to oxidative stress. We also investigated the levels of iNOS and Nrf2 to evaluate if α-tocopherol was able to counteract oxidative stress. We observed that Aβ treatment increased oxidative stress, as demonstrated by the increased iNOS levels, while Nrf2 was reduced. On the contrary, α-tocopherol restored Nrf2 levels and inhibited iNOS. Interestingly, the genes encoding for subunits of the V-type ATPase (*ATP6V1C1* and *ATP6V1H*) were upregulated, with only *ATP6V0A2* downregulated. V-type ATPase is a complex that acts as an ATP-dependent proton pump found in the endomembrane system, where it is involved in the acidification of the lumen of subcellular organelles such as lysosomes and endosomes. The V-type ATPase function is necessary for different actions other than pH, indeed it is even necessary for the formation of synaptic vesicles [49]. Data showed the involvement of V-ATPase deficiency in the pathogenesis of AD [50].

The apoptotic peptidase activating factor 1 (APAF1) has a main role in inducing apoptosis, taking part in apoptosome formation [51]. The treatment with α-tocopherol was able to reduce *APAF1* expression, indicating a reduction of apoptosis.

AD is characterized by an abnormal cell cycle re-entry of neurons, which may have a causal role in neurodegeneration. AD neurons showed high levels of the cell cycle markers, such as cyclins and cyclin-dependent kinases (CDK), and markers of DNA replication [52]. In particular, it was reported that Aβ was able to activate the cell cycle in SH-SY5Y [53]. The cell cycle re-entry seems to lead to apoptosis in neurological diseases. In particular, oxidative stress may be responsible for cell cycle re-entry. Indeed, oxidative stress may induce DNA damage with the consequent activation of enzymes involved in DNA repair. In turn, they can modulate downstream targets that regulate the proteins involved in the cell cycle checkpoint, leading at the end to cell cycle re-entry [54]. On the contrary, α-tocopherol treatment was able to reduce the expression of cyclins D, A, and B, which are encoded by *CCND1*, *CCNA2*, *CCNB1* and *CCNB2* respectively, together with BUB1 mitotic checkpoint serine/threonine kinase B (*BUB1B)*, BUB3 mitotic checkpoint protein (*BUB3)*, minichromosome maintenance complex component 4 (*MCM4),* minichromosome maintenance complex component 6 (*MCM6),* and Polo like kinase 1 (*PLK1),* compared to cells treated with Aβ. PLK1 acts during mitosis from G2 until the last phase of cytokinesis [55]. The proteins MCM4 and MCM6 are part of a heterohexameric complex formed by MCM2-MCM7, which plays a role as a replicative DNA helicase during DNA replication [56]. BubR1, together with Bub3, is part of the spindle assembly checkpoint that acts in preventing the loss of sister chromatid cohesion and premature chromosome segregation in the presence of unattached or incorrectly attached chromosomes [57]. In association, also mitotic arrest deficient 2 like 1 (*MAD2L1)*, encoding for MAD2—which is part of the spindle assembly checkpoint together with BubR1 and Bub3—was significantly reduced in cells treated with α-tocopherol and Aβ. *CDK1*, encoding for CDK1, was also significantly reduced in cells treated with α-tocopherol and Aβ. CDKs are protein kinases that are associated with cyclins’ control cell cycle [58]. Anaphase-promoting complex/cyclosome (APC/C) is responsible for the destruction of cyclins and other cell cycle proteins, and promotes anaphase [59]. We observed the upregulation of the genes encoded for APC/C (*CDC27*, *CDC16*), while only *ANAPC1* was downregulated. APC/C was reported to be downregulated and inactivated in AD, probably causing the aberrant cycle re-entry [60]. Also, the genes encoding for securin and structural maintenance of chromosomes 1A, *PTTG1* and *SMC1A* respectively, were downregulated in SH-SY5Y cells treated with α-tocopherol and Aβ. Cell Division Cycle-25 (CDC25) acts through dephosphorylating and activating CDKs, and for this reason, it has a main role in the transition between the phases of the cell cycle. We found the downregulation of *CDC25B*, which is involved in G2/M progression [61]. In parallel, we observed the upregulation of the genes encoding for 14-3-3 proteins (*YWHAE, YWHAQ, YWHAZ, YWHAG*) in cells treated with α-tocopherol and Aβ. 14-3-3 proteins inhibited CDC25 activity [62]. Transcriptional analysis revealed the upregulation of *WEE1* in SH-SY5Y cells treated with α-tocopherol and Aβ compared to those exposed to Aβ. The Wee protein phosphorylated, causing the inhibition of CDK1 [63]. *ABL1*, which plays a role in spindle orientation control, was reduced, while *SKP1*, which is part of the SKP1-Cullin 1-F-box protein (SCF) complex [64], was increased.

It was demonstrated that the abnormal re-entry of neurons into the cell cycle is linked to changes of both mitochondria and autophagy, and then to neuronal degeneration. Neuroblastoma cells treated with an inhibitor of autophagy showed that the proportion of cells in G2/M was significantly increased, while the number of cells in G0/G1 was reduced. In parallel, autophagic proteins were accumulated in association with the changes in proteins responsible for the mitochondrial cycle [65].

Dysfunction of the autophagy is involved in different neurodegenerative disorders, including AD [12,13]. The autophagosomes in AD brains may be a reservoir of Aβ; then, enhancing the formation of new autophagosomes without the associated increase in their degradation may cause an increase in Aβ production, accumulation, and as a consequence, its toxicity [13].

The process of autophagy is composed by different phases: the initiation and nucleation of the phagophore, its expansion and autophagophore formation, the fusion of the autophagosome with lysosomes, and the final degradation in autolysosomes [66]. In healthy neurons, autophagosomes are scarce thanks to the efficiency of the autophagic flux, while in AD, the accumulation of autophagic vacuoles was observed [13]. Different approaches that induce the upregulation of autophagy were reported to be potential therapeutic strategies against AD [12,67]. Lipinski et al. found the transcriptional upregulation of autophagy in the brains of AD patients, speculating about a possible compensatory regulation of autophagy [68]. Moreover, they found an increased initiation of autophagy in response to Aβ and a suppression of lysosomal proteolysis [68].

The combination of increased autophagy induction and damaged clearance of autophagic vacuoles represents a favorable condition to cause Aβ accumulation in AD [69]. The excessive induction of autophagy beyond the clearance capability of the lysosome led to the accumulation of autophagosomes in the neurons, leading to toxicity [12,70].

α-tocopherol treatment downregulated unc-51 like autophagy activating kinase 2 (*ULK2)*, whose protein seems to have a redundant function with Unc-51 Like Autophagy Activating Kinase 1 (ULK1), which is part of a complex that has a role in autophagy initiation [71]. Moreover, we found the modulation of different genes whose encoded proteins act as regulators of the phosphoinositide-3-kinase (PI3K) complex of autophagy. In particular, vacuole membrane protein 1 (*VMP1)* and syntaxin 17 (*STX17)* were downregulated, while UV radiation resistance associated (*UVRAG)* and autophagy and beclin 1 regulator 1 (*AMBRA1)* were upregulated. The protein VMP1 is needed for autophagosome formation and autophagy induction [72]. STX17 is required for the autophagosome maturation phase [73]. AMBRA1 is needed for Beclin1 translocation to the ER and the subsequent induction of autophagy [72]. UVRAG has a role in autophagosome formation and maturation [72]. Myotubularin related protein 3 (*MTMR3)* and WD repeat domain, phosphoinositide interacting 1 *(WIPI1)* were upregulated, while WD repeat domain, phosphoinositide interacting 2 (*WIPI2*) was downregulated. The role of the protein encoded by *MTMR3* was to inhibit autophagy through the dephosphorylation of phosphatidylinositol 3-phosphate (PI3P), which has a role in autophagosome formation [74]. The WIPI proteins interact with PI3P and play a role as PI3P effector proteins, recruiting downstream autophagy-related (ATG) proteins [75]. This data may indicate the induction of autophagy, even if the downregulation of some genes may limit the excessive autophagic flux. Also, *GABARAP* and *GABARAPL1*, encoding for members of the microtubule-associated protein 1A/1B-light chain 3 (LC3) family [76], and *ATG3* were upregulated. Cleaved LC3 is conjugated to phosphatidylethanolamine (PE) by the sequential activation of Atg7 (E1-like enzyme), Atg3 (E2-like enzyme), and the Atg12 complex, to generate LC3-PE (a membrane-bound form of LC3), the level of which is correlated with the number of autophagosomes. In addition, during selective autophagy, LC3 acts as an adaptor protein to recruit selective cargo to the autophagosome via interaction with cargo receptors [77]. *ATG4A* and *ATG4B* were downregulated. Processing and delipidation reactions of LC3 by Atg4 are required for autophagosome formation [78]. Moreover, the genes *CTSB* and *CTSD* encoding for cathepsins were upregulated. On the contrary, the downregulation of *CTSL* was found, which encoded for cathepsin L, and is involved in the degradation of the autophagosomal membrane markers, such as GABARAP and LC3—in contrast to the other cathepsins, which are involved in the degradation of the content of autophagosomes [79]. The increased activity of cathepsins is in line with the results regarding the V-type ATPases, which is required to maintain the acidic pH in the lysosome that is needed for the functioning of lysosomal enzymes. These results may indicate a better degradation of autophagosome cargo, but the proteins involved in the autophagic flux are not degraded. Given that the literature data indicated the increase of autophagy initiation and the accumulation of autophagosomes in AD, our results may indicate that α-tocopherol is able to increase the degradation of the formed autophagosomes, leading to a clearance of Aβ.

## 5. Conclusions

In conclusion, our results suggested that α-tocopherol was able to counteract Aβ toxicity, inducing the non-amyloidogenic processing pathway of APP and modulating the autophagic flux in order to induce the degradation of the formed autophagosomes. Moreover, α-tocopherol inhibited the cell cycle that is known to be abnormally reactivated in AD. This data indicated that α-tocopherol was able to modulate pathways that are altered in AD, suggesting that it may be useful in AD treatment.

## Figures and Tables

**Table 1:** Genes significantly affected by amyloid β (Aβ) and α-tocopherol involved in the Alzheimer’s disease pathway. Each gene in the first column was associated with the expression value in each group, the fold change was computed as the Log_2_(SH-SY5Y-Aβ versus SH-SY5Y-Aβ-αT) and the name of the related protein in KEGG. The values were rounded to the second decimal digit.

Gene	Expression Level SH-SY5Y-Aβ	Expression Level SH-SY5Y-Aβ-αT	Fold Change SH-SY5Y-Aβ vs SH-SY5Y-Aβ-αT	KEGG
*APP*	790.97	831.23	0.07	APP
*ADAM17*	43.75	51.53	0.24	ADAM17
*RTN4*	145.96	172.05	0.24	RTN4
*PSEN2*	36.60	27.17	−0.43	PSEN
*APH1B*	0.71	4.27	2.59	APH-1
*NDUFB1*	315.96	386.93	0.29	Cx I
*NDUFB3*	33.35	45.25	0.44	Cx I
*NDUFC2*	125.39	157.85	0.33	Cx I
*NDUFS4*	110.35	134.55	0.29	Cx I
*NDUFA1*	140.33	116.04	−0.27	Cx I
*NDUFA6*	44.59	27.70	−0.69	Cx I
*SDHA*	90.76	72.85	−0.32	Cx II
*UQCRB*	319.48	465.93	0.54	Cx III
*UQCRH*	409.51	459.12	0.17	Cx III
*COX7A2*	317.64	368.03	0.21	Cx IV
*APAF1*	32.15	23.13	−0.48	Apaf1

*APP*, amyloid precursor protein; *ADAM17*, ADAM metallopeptidase domain 17; *RTN4*, Reticulon 4; *PSEN2*, presenilin 2; *APH1B*, aph-1 homolog B, gamma-secretase subunit; *NDUFB1*, NADH:ubiquinone oxidoreductase subunit B1; *NDUFB3*, NADH:ubiquinone oxidoreductase subunit B3; *NDUFC2*, NADH:ubiquinone oxidoreductase subunit C2; *NDUFS4*, NADH:ubiquinone oxidoreductase subunit S4; *NDUFA1*, NADH:ubiquinone oxidoreductase subunit A1; *NDUFA6*, NADH:ubiquinone oxidoreductase subunit A6; *SDHA*, succinate dehydrogenase complex flavoprotein subunit A; *UQCRB*, ubiquinol-cytochrome c reductase binding protein; *UQCRH*, ubiquinol-cytochrome c reductase hinge protein; *COX7A2*, cytochrome c oxidase subunit 7A2; *APAF1*, apoptotic peptidase activating factor 1.

**Table 2:** Genes significantly affected by Aβ and α-tocopherol involved in the oxidative phosphorylation pathway. Each gene in the first column was associated with the expression value in each group, the fold change was computed as the Log_2_(SH-SY5Y-Aβ versus SH-SY5Y-Aβ-αT) and the name of the related protein in KEGG. The values were rounded to the second decimal digit.

Gene	Expression Level SH-SY5Y-Aβ	Expression Level SH-SY5Y-Aβ-αT	Fold Change SH-SY5Y-Aβ vs SH-SY5Y-Aβ-αT	KEGG
*NDUFB1*	315.96	386.93	0.29	Cx I
*NDUFB3*	33.35	45.25	0.44	Cx I
*NDUFC2*	125.39	157.85	0.33	Cx I
*NDUFS4*	110.35	134.55	0.29	Cx I
*NDUFA1*	140.33	116.04	−0.27	Cx I
*NDUFA6*	44.59	27.70	−0.69	Cx I
*SDHA*	90.76	72.85	−0.32	Cx II
*UQCRB*	319.48	465.93	0.54	Cx III
*UQCRH*	409.51	459.12	0.17	Cx III
*COX7A2*	317.64	368.03	0.21	Cx IV
*COX10*	21.36	30.59	0.52	Cx IV
*ATP6V0A2*	107.86	83.99	−0.36	V-type ATPase
*ATP6V1C1*	31.00	47.08	0.60	V-type ATPase
*ATP6V1H*	17.21	28.31	0.72	V-type ATPase

*NDUFB1*, NADH:ubiquinone oxidoreductase subunit B1; *NDUFB3*, NADH:ubiquinone oxidoreductase subunit B3; NDUFC2, NADH:ubiquinone oxidoreductase subunit C2; *NDUFS4*, NADH:ubiquinone oxidoreductase subunit S4; *NDUFA1*, NADH:ubiquinone oxidoreductase subunit A1; *NDUFA6*, NADH:ubiquinone oxidoreductase subunit A6; *SDHA*, succinate dehydrogenase complex flavoprotein subunit A; *UQCRB*, ubiquinol-cytochrome c reductase binding protein; *UQCRH*, ubiquinol-cytochrome c reductase hinge protein; *COX7A2*, cytochrome c oxidase subunit 7A2; *COX10*, cytochrome c oxidase assembly factor heme A:farnesyltransferase COX10; *ATP6V0A2*, ATPase H+ transporting V0 subunit a2; *ATP6V1C1*, ATPase H+ transporting V1 subunit C1; *ATP6V1H*, ATPase H+ transporting V1 subunit H.

**Table 3:** Genes significantly affected by Aβ and α-tocopherol involved in the cell cycle pathway. Each gene in the first column was associated with the expression value in each group, the fold change was computed as the Log_2_(SH-SY5Y-Aβ versus SH-SY5Y-Aβ-αT) and the name of the related protein in KEGG. The values were rounded to the second decimal digit.

Gene	Expression Level SH-SY5Y-Aβ	Expression Level SH-SY5Y-Aβ-αT	Fold Change SH-SY5Y-Aβ vs SH-SY5Y-Aβ-αT	KEGG
*SMC1A*	308.34	265.09	−0.22	Smc1
*PTTG1*	238.77	195.25	−0.29	PTTG
*ANAPC1*	27.67	21.81	−0.34	APC/C
*CDC27*	72.41	96.18	0.41	APC/C
*CDC16*	47.73	56.45	0.24	APC/C
*MAD2L1*	41.47	20.97	−0.98	Mad2
*BUB1B*	192.20	162.39	−0.24	BubR1
*BUB3*	108.93	85.13	−0.36	Bub3
*YWHAE*	839.28	920.19	0.13	14-3-3
*YWHAQ*	528.10	617.89	0.23	14-3-3
*YWHAZ*	337.73	382.05	0.18	14-3-3
*YWHAG*	54.03	59.33	0.14	14-3-3
*CDC25B*	22.56	16.98	−0.41	Cdc25B
*PLK1*	75.39	44.80	−0.75	Plk1
*CCNB1*	115.68	82.62	−0.49	CycB
*CCNB2*	32.32	24.88	−0.38	CycB
*CDK1*	55.86	40.01	−0.48	CDK1
*CCNA2*	28.88	24.31	−0.25	CycA
*WEE1*	54.85	69.70	0.35	Wee
*MCM4*	424.40	327.06	−0.38	MCM
*MCM6*	42.88	36.64	−0.23	MCM
*ABL1*	26.96	22.39	−0.27	Abl
*CCND1*	326.55	269.68	−0.28	CycD
*SKP1*	505.53	563.13	0.16	SCF

*SMC1A*, structural maintenance of chromosomes 1A; *PTTG1*, regulator of sister chromatid separation, securin; *ANAPC1*, anaphase promoting complex subunit 1; *CDC27*, cell division cycle 27; *CDC16*, cell division cycle 16; *MAD2L1*, mitotic arrest deficient 2 like 1; *BUB1B*, BUB1 mitotic checkpoint serine/threonine kinase B; *BUB3*, BUB3 mitotic checkpoint protein; *YWHAE*, tyrosine 3-monooxygenase/tryptophan 5-monooxygenase activation protein epsilon; *YWHAQ*, tyrosine 3-monooxygenase/tryptophan 5-monooxygenase activation protein theta; *YWHAZ*, tyrosine 3-monooxygenase/tryptophan 5-monooxygenase activation protein zeta; *YWHAG*, tyrosine 3-monooxygenase/tryptophan 5-monooxygenase activation protein gamma; *CDC25B*, cell division cycle 25B; *PLK1*, polo like kinase 1; *CCNB1*, cyclin B1; *CCNB2*, cyclin B2; *CDK1*, cyclin dependent kinase 1; *CCNA2*, cyclin A2; *WEE1*, WEE1 G2 checkpoint kinase; *MCM4*, minichromosome maintenance complex component 4; *MCM6*, minichromosome maintenance complex component 6; *ABL1*, ABL proto-oncogene 1, non-receptor tyrosine kinase; *CCND1*, cyclin D1; *SKP1*, S-phase kinase associated protein 1.

**Table 4:** Genes significantly affected by Aβ and α-tocopherol involved in the autophagy–animal pathway. To each gene in the first column, the expression value in each group, the fold change that was computed as the Log_2_(SH-SY5Y-Aβ versus SH-SY5Y-Aβ-αT) and the name of the related protein in KEGG were associated. The values were rounded to the second decimal digit.

Gene	Expression Level SH-SY5Y-Aβ	Expression Level SH-SY5Y-Aβ-αT	Fold Change SH-SY5Y-Aβ vs SH-SY5Y-Aβ-αT	KEGG
*CTSB*	29.90	46.25	0.63	CTSB
*CTSL*	25.55	17.53	−0.54	CTSL
*CTSD*	39.26	51.95	0.40	CTSD
*STX17*	34.35	17.14	−1.00	STX17
*GABARAP*	322.29	387.87	0.27	LC3
*GABARAPL1*	10.63	19.36	0.87	LC3
*ATG3*	98.92	126.93	0.36	ATG3
*ATG4A*	8.14	3.51	−1.21	ATG4
*ATG4B*	65.88	43.33	−0.61	ATG4
*MTMR3*	27.34	38.55	0.50	MRMR3
*UVRAG*	9.34	16.50	0.82	UVRAG
*AMBRA1*	13.36	17.45	0.39	AMBRA1
*VMP1*	72.46	57.04	−0.35	VMP1
*WIPI1*	9.11	15.35	0.75	WIPI
*WIPI2*	58.48	45.25	−0.37	WIPI
*ULK2*	58.01	37.97	−0.61	ULK2

*CTSB*, Cathepsin B; *CTSL*, cathepsin L; *CTSD*, cathepsin D; *STX17*, syntaxin 17; *GABARAP*, GABA type A receptor-associated protein; *GABARAPL1*, GABA type A receptor associated protein like 1; *ATG3*, autophagy related 3; *ATG4A*, autophagy related 4A cysteine peptidase; *ATG4B*, autophagy related 4B cysteine peptidase; *MTMR3*, myotubularin related protein 3; *UVRAG*, UV radiation resistance associated; *AMBRA1*, autophagy and beclin 1 regulator 1; *VMP1*, vacuole membrane protein 1; *WIPI1*, WD repeat domain, phosphoinositide interacting 1; *WIPI2*, WD repeat domain, phosphoinositide interacting 2; *ULK2*, unc-51 like autophagy activating kinase 2.

## References

[1] Zverova M. (2019). Clinical aspects of Alzheimer’s disease. Clin. Biochem..

[2] Fiscon G., Weitschek E., Cialini A., Felici G., Bertolazzi P., De Salvo S., Bramanti A., Bramanti P., De Cola M.C. (2018). Combining eeg signal processing with supervised methods for Alzheimer’s patients classification. BMC Med. Inform. Decis. Mak..

[3] Lo Buono V., Bonanno L., Corallo F., Foti M., Palmeri R., Marra A., Di Lorenzo G., Todaro A., Bramanti P., Bramanti A. (2018). Qualitative analysis of mini mental state examination pentagon in vascular dementia and alzheimer’s disease: A longitudinal explorative study. J. Stroke Cerebrovasc. Dis..

[4] Allone C., Lo Buono V., Corallo F., Bonanno L., Palmeri R., Di Lorenzo G., Marra A., Bramanti P., Marino S. (2018). Cognitive impairment in Parkinson’s disease, Alzheimer’s dementia, and vascular dementia: The role of the clock-drawing test. Psychogeriatrics.

[5] Yuksel M., Tacal O. (2019). Trafficking and proteolytic processing of amyloid precursor protein and secretases in alzheimer’s disease development: An up-to-date review. Eur. J. Pharmacol..

[6] Qiu T., Liu Q., Chen Y.X., Zhao Y.F., Li Y.M. (2015). Abeta42 and abeta40: Similarities and differences. J. Pept. Sci. Off. Publ. Eur. Pept. Soc..

[7] Butterfield D.A., Swomley A.M., Sultana R. (2013). Amyloid beta-peptide (1-42)-induced oxidative stress in alzheimer disease: Importance in disease pathogenesis and progression. Antioxid. Redox Signal..

[8] Gao Y., Tan L., Yu J.T., Tan L. (2018). Tau in Alzheimer’s disease: Mechanisms and therapeutic strategies. Curr. Alzheimer Res..

[9] Penke B., Bogar F., Fulop L. (2017). Beta-amyloid and the pathomechanisms of alzheimer’s disease: A comprehensive view. Molecules.

[10] Ardura-Fabregat A., Boddeke E., Boza-Serrano A., Brioschi S., Castro-Gomez S., Ceyzeriat K., Dansokho C., Dierkes T., Gelders G., Heneka M.T. (2017). Targeting neuroinflammation to treat Alzheimer’s disease. CNS Drugs.

[11] Bostanciklioglu M. (2019). An update on the interactions between Alzheimer’s disease, autophagy and inflammation. Gene.

[12] Meng T., Lin S., Zhuang H., Huang H., He Z., Hu Y., Gong Q., Feng D. (2019). Recent progress in the role of autophagy in neurological diseases. Cell Stress.

[13] Ntsapi C., Lumkwana D., Swart C., du Toit A., Loos B. (2018). New insights into autophagy dysfunction related to amyloid beta toxicity and neuropathology in alzheimer’s disease. Int. Rev. Cell Mol. Biol..

[14] Dong Y., Chen X., Liu Y., Shu Y., Chen T., Xu L., Li M., Guan X. (2018). Do low-serum vitamin e levels increase the risk of alzheimer disease in older people? Evidence from a meta-analysis of case-control studies. Int. J. Geriatr. Psychiatry.

[15] Jimenez-Jimenez F.J., de Bustos F., Molina J.A., Benito-Leon J., Tallon-Barranco A., Gasalla T., Orti-Pareja M., Guillamon F., Rubio J.C., Arenas J. (1997). Cerebrospinal fluid levels of alpha-tocopherol (vitamin e) in Alzheimer’s disease. J. Neural Transm..

[16] Mangialasche F., Kivipelto M., Mecocci P., Rizzuto D., Palmer K., Winblad B., Fratiglioni L. (2010). High plasma levels of vitamin e forms and reduced Aalzheimer’s disease risk in advanced age. J. Alzheimer’s Dis. JAD.

[17] Galli F., Azzi A., Birringer M., Cook-Mills J.M., Eggersdorfer M., Frank J., Cruciani G., Lorkowski S., Ozer N.K. (2017). Vitamin e: Emerging aspects and new directions. Free Radic. Biol. Med..

[18] Zingg J.M. (2019). Vitamin e: Regulatory role on signal transduction. IUBMB Life.

[19] Niki E. (2014). Role of vitamin e as a lipid-soluble peroxyl radical scavenger: In vitro and in vivo evidence. Free Radic. Biol. Med..

[20] Ulatowski L., Parker R., Warrier G., Sultana R., Butterfield D.A., Manor D. (2014). Vitamin E is essential for purkinje neuron integrity. Neuroscience.

[21] Gugliandolo A., Bramanti P., Mazzon E. (2017). Role of vitamin e in the treatment of Alzheimer’s disease: Evidence from animal models. Int. J. Mol. Sci..

[22] Lloret A., Esteve D., Monllor P., Cervera-Ferri A., Lloret A. (2019). The effectiveness of vitamin E treatment in Alzheimer’s disease. Int. J. Mol. Sci..

[23] Yang S.G., Wang W.Y., Ling T.J., Feng Y., Du X.T., Zhang X., Sun X.X., Zhao M., Xue D., Yang Y. (2010). Alpha-tocopherol quinone inhibits beta-amyloid aggregation and cytotoxicity, disaggregates preformed fibrils and decreases the production of reactive oxygen species, no and inflammatory cytokines. Neurochem. Int..

[24] Castillo W.O., Aristizabal-Pachon A.F., de Lima Montaldi A.P., Sakamoto-Hojo E.T., Takahashi C.S. (2016). Galanthamine decreases genotoxicity and cell death induced by beta-amyloid peptide in sh-sy5y cell line. Neurotoxicology.

[25] Castillo W.O., Aristizabal-Pachon A.F., Sakamoto-Hojo E., Gasca C.A., Cabezas-Fajardo F.A., Takahashi C. (2018). Caliphruria subedentata (amaryllidaceae) decreases genotoxicity and cell death induced by beta-amyloid peptide in sh-sy5y cell line. Mutat. Res. Genet. Toxicol. Environ. Mutagenesis.

[26] Mei Z., Yan P., Situ B., Mou Y., Liu P. (2012). Cryptotanshinione inhibits beta-amyloid aggregation and protects damage from beta-amyloid in sh-sy5y cells. Neurochem. Res..

[27] Seino S., Kimoto T., Yoshida H., Tanji K., Matsumiya T., Hayakari R., Seya K., Kawaguchi S., Tsuruga K., Tanaka H. (2018). Gnetin c, a resveratrol dimer, reduces amyloid-beta 1-42 (abeta42) production and ameliorates abeta42-lowered cell viability in cultured sh-sy5y human neuroblastoma cells. Biomed. Res..

[28] Chiricosta L., Diomede F., Trubiani O., Bramanti P., Mazzon E. (2019). Physiological expression of ion channel receptors in human periodontal ligament stem cells. Cells.

[29] Bolger A.M., Lohse M., Usadel B. (2014). Trimmomatic: A flexible trimmer for illumina sequence data. Bioinformatics.

[30] University of California Santa Cruz (Ucsc). http://labshare.cshl.edu/shares/gingeraslab/www-data/dobin/STAR/STARgenomes/ENSEMBL/homo_sapiens/ENSEMBL.homo_sapiens.release-75/Homo_sapiens.GRCh37.75.dna.primary_assembly.fa.

[31] Dobin A., Davis C.A., Schlesinger F., Drenkow J., Zaleski C., Jha S., Batut P., Chaisson M., Gingeras T.R. (2013). Star: Ultrafast universal rna-seq aligner. Bioinformatics.

[32] Trapnell C., Hendrickson D.G., Sauvageau M., Goff L., Rinn J.L., Pachter L. (2013). Differential analysis of gene regulation at transcript resolution with rna-seq. Nat. Biotechnol..

[33] Kanehisa M., Goto S. (2000). Kegg: Kyoto encyclopedia of genes and genomes. Nucleic Acids Res..

[34] Kyoto encyclopedia of genes and genomes (kegg). https://www.genome.jp/kegg/pathway.html.

[35] Cervantes B., Ulatowski L.M. (2017). Vitamin E and Alzheimer’s disease-is it time for personalized medicine?. Antioxidants.

[36] La Fata G., Weber P., Mohajeri M.H. (2014). Effects of vitamin e on cognitive performance during ageing and in alzheimer’s disease. Nutrients.

[37] Meng P., Yoshida H., Matsumiya T., Imaizumi T., Tanji K., Xing F., Hayakari R., Dempoya J., Tatsuta T., Aizawa-Yashiro T. (2013). Carnosic acid suppresses the production of amyloid-beta 1-42 by inducing the metalloprotease gene tace/adam17 in sh-sy5y human neuroblastoma cells. Neurosci. Res..

[38] Murayama K.S., Kametani F., Saito S., Kume H., Akiyama H., Araki W. (2006). Reticulons rtn3 and rtn4-b/c interact with bace1 and inhibit its ability to produce amyloid beta-protein. Eur. J. Neurosci..

[39] He W., Lu Y., Qahwash I., Hu X.Y., Chang A., Yan R. (2004). Reticulon family members modulate bace1 activity and amyloid-beta peptide generation. Nat. Med..

[40] Checler F., Dunys J., Pardossi-Piquard R., Alves da Costa C. (2010). P53 is regulated by and regulates members of the gamma-secretase complex. Neuro Degener. Dis..

[41] Dunys J., Kawarai T., Sevalle J., Dolcini V., George-Hyslop P.S., Da Costa C.A., Checler F. (2007). P53-dependent aph-1 and pen-2 anti-apoptotic phenotype requires the integrity of the gamma-secretase complex but is independent of its activity. J. Biol. Chem..

[42] Zhou Z.D., Chan C.H., Ma Q.H., Xu X.H., Xiao Z.C., Tan E.K. (2011). The roles of amyloid precursor protein (app) in neurogenesis: Implications to pathogenesis and therapy of Alzheimer disease. Cell Adhes. Migr..

[43] Ryan M.M., Morris G.P., Mockett B.G., Bourne K., Abraham W.C., Tate W.P., Williams J.M. (2013). Time-dependent changes in gene expression induced by secreted amyloid precursor protein-alpha in the rat hippocampus. BMC Genom..

[44] Chasseigneaux S., Allinquant B. (2012). Functions of abeta, sappalpha and sappbeta: Similarities and differences. J. Neurochem..

[45] Wang Y.Q., Qu D.H., Wang K. (2016). Therapeutic approaches to Alzheimer’s disease through stimulating of non-amyloidogenic processing of amyloid precursor protein. Eur. Rev. Med Pharmacol. Sci..

[46] Perez Ortiz J.M., Swerdlow R.H. (2019). Mitochondrial dysfunction in Alzheimer’s disease: Role in pathogenesis and novel therapeutic opportunities. Br. J. Pharmacol..

[47] Manczak M., Anekonda T.S., Henson E., Park B.S., Quinn J., Reddy P.H. (2006). Mitochondria are a direct site of a beta accumulation in alzheimer’s disease neurons: Implications for free radical generation and oxidative damage in disease progression. Hum. Mol. Genet..

[48] Kawamata H., Manfredi G. (2017). Proteinopathies and oxphos dysfunction in neurodegenerative diseases. J. Cell Biol..

[49] Pamarthy S., Kulshrestha A., Katara G.K., Beaman K.D. (2018). The curious case of vacuolar atpase: Regulation of signaling pathways. Mol. Cancer.

[50] Colacurcio D.J., Nixon R.A. (2016). Disorders of lysosomal acidification-the emerging role of v-atpase in aging and neurodegenerative disease. Ageing Res. Rev..

[51] Green D.R. (2005). Apoptotic pathways: Ten minutes to dead. Cell.

[52] Bonda D.J., Lee H.P., Kudo W., Zhu X., Smith M.A., Lee H.G. (2010). Pathological implications of cell cycle re-entry in Alzheimer disease. Expert Rev. Mol. Med..

[53] Frasca G., Chiechio S., Vancheri C., Nicoletti F., Copani A., Angela Sortino M. (2004). Beta-amyloid-activated cell cycle in sh-sy5y neuroblastoma cells: Correlation with the map kinase pathway. J. Mol. Neurosci. MN.

[54] Folch J., Junyent F., Verdaguer E., Auladell C., Pizarro J.G., Beas-Zarate C., Pallas M., Camins A. (2012). Role of cell cycle re-entry in neurons: A common apoptotic mechanism of neuronal cell death. Neurotox. Res..

[55] Colicino E.G., Hehnly H. (2018). Regulating a key mitotic regulator, polo-like kinase 1 (plk1). Cytoskeleton.

[56] Ishimi Y. (2018). Regulation of mcm2-7 function. Genes Genet. Syst..

[57] Musacchio A. (2015). The molecular biology of spindle assembly checkpoint signaling dynamics. Curr. Biol. CB.

[58] Malumbres M. (2014). Cyclin-dependent kinases. Genome Biol..

[59] Kernan J., Bonacci T., Emanuele M.J. (2018). Who guards the guardian? Mechanisms that restrain apc/c during the cell cycle. Biochim. Biophys. Acta Mol. Cell Res..

[60] Fuchsberger T., Lloret A., Vina J. (2017). New functions of apc/c ubiquitin ligase in the nervous system and its role in alzheimer’s disease. Int. J. Mol. Sci..

[61] Sur S., Agrawal D.K. (2016). Phosphatases and kinases regulating cdc25 activity in the cell cycle: Clinical implications of cdc25 overexpression and potential treatment strategies. Mol. Cell. Biochem..

[62] Gardino A.K., Yaffe M.B. (2011). 14-3-3 proteins as signaling integration points for cell cycle control and apoptosis. Semin. Cell Dev. Biol..

[63] Schmidt M., Rohe A., Platzer C., Najjar A., Erdmann F., Sippl W. (2017). Regulation of g2/m transition by inhibition of wee1 and pkmyt1 kinases. Molecules.

[64] Zheng N., Zhou Q., Wang Z., Wei W. (2016). Recent advances in scf ubiquitin ligase complex: Clinical implications. Biochim. Biophys. Acta.

[65] Gui M.C., Chen B., Yu S.S., Bu B.T. (2014). Effects of suppressed autophagy on mitochondrial dynamics and cell cycle of n2a cells. J. Huazhong Univ. Sci. Technol. Med.

[66] Cheng Y., Ren X., Hait W.N., Yang J.M. (2013). Therapeutic targeting of autophagy in disease: Biology and pharmacology. Pharmacol. Rev..

[67] Uddin M.S., Stachowiak A., Mamun A.A., Tzvetkov N.T., Takeda S., Atanasov A.G., Bergantin L.B., Abdel-Daim M.M., Stankiewicz A.M. (2018). Autophagy and Alzheimer’s disease: From molecular mechanisms to therapeutic implications. Front. Aging Neurosci..

[68] Lipinski M.M., Zheng B., Lu T., Yan Z., Py B.F., Ng A., Xavier R.J., Li C., Yankner B.A., Scherzer C.R. (2010). Genome-wide analysis reveals mechanisms modulating autophagy in normal brain aging and in Alzheimer’s disease. Proc. Natl. Acad. Sci. USA.

[69] Nixon R.A. (2007). Autophagy, amyloidogenesis and Alzheimer disease. J. Cell Sci..

[70] Boland B., Kumar A., Lee S., Platt F.M., Wegiel J., Yu W.H., Nixon R.A. (2008). Autophagy induction and autophagosome clearance in neurons: Relationship to autophagic pathology in Alzheimer’s disease. J. Neurosci..

[71] Zachari M., Ganley I.G. (2017). The mammalian ulk1 complex and autophagy initiation. Essays Biochem..

[72] Kang R., Zeh H.J., Lotze M.T., Tang D. (2011). The beclin 1 network regulates autophagy and apoptosis. Cell Death Differ..

[73] Viret C., Faure M. (2019). Regulation of syntaxin 17 during autophagosome maturation. Trends Cell Biol..

[74] Vergne I., Deretic V. (2010). The role of pi3p phosphatases in the regulation of autophagy. FEBS Lett..

[75] Grimmel M., Backhaus C., Proikas-Cezanne T. (2015). Wipi-mediated autophagy and longevity. Cells.

[76] Schaaf M.B., Keulers T.G., Vooijs M.A., Rouschop K.M. (2016). Lc3/gabarap family proteins: Autophagy-(un)related functions. FASEB J..

[77] Lee Y.K., Lee J.A. (2016). Role of the mammalian atg8/lc3 family in autophagy: Differential and compensatory roles in the spatiotemporal regulation of autophagy. BMB Rep..

[78] Maruyama T., Noda N.N. (2017). Autophagy-regulating protease atg4: Structure, function, regulation and inhibition. J. Antibiot..

[79] Kaminskyy V., Zhivotovsky B. (2012). Proteases in autophagy. Biochim. Biophys. Acta.

